# Identification of Resting-State Network Functional Connectivity and Brain Structural Signatures in Fibromyalgia Using a Machine Learning Approach

**DOI:** 10.3390/biomedicines10123002

**Published:** 2022-11-22

**Authors:** Nguyen Thanh Nhu, David Yen-Ting Chen, Jiunn-Horng Kang

**Affiliations:** 1 International Ph.D. Program in Medicine, College of Medicine, Taipei Medical University, Taipei 110, Taiwan; 2 Faculty of Medicine, Can Tho University of Medicine and Pharmacy, Can Tho 94117, Vietnam; 3 Department of Medical Imaging, Taipei Medical University-Shuang-Ho Hospital, New Taipei City 235, Taiwan; 4 Department of Radiology, School of Medicine, College of Medicine, Taipei Medical University, Taipei 110, Taiwan; 5 Department of Physical Medicine and Rehabilitation, School of Medicine, College of Medicine, Taipei Medical University, Taipei 110, Taiwan; 6 Department of Physical Medicine and Rehabilitation, Taipei Medical University Hospital, Taipei 110, Taiwan; 7 Graduate Institute of Nanomedicine and Medical Engineering, College of Biomedical Engineering, Taipei Medical University, Taipei 110, Taiwan

**Keywords:** fibromyalgia, functional connectivity, brain structure, MRI, machine learning

## Abstract

Abnormal resting-state functional connectivity (rs-FC) and brain structure have emerged as pathological hallmarks of fibromyalgia (FM). This study investigated and compared the accuracy of network rs-FC and brain structural features in identifying FM with a machine learning (ML) approach. Twenty-six FM patients and thirty healthy controls were recruited. Clinical presentation was measured by questionnaires. After MRI acquisitions, network rs-FC z-score and network-based gray matter volume matrices were exacted and preprocessed. The performance of feature selection and classification methods was measured. Correlation analyses between predictive features in final models and clinical data were performed. The combination of the recursive feature elimination (RFE) selection method and support vector machine (rs-FC data) or logistic regression (structural data), after permutation importance feature selection, showed high performance in distinguishing FM patients from pain-free controls, in which the rs-FC ML model outperformed the structural ML model (accuracy: 0.91 vs. 0.86, AUC: 0.93 vs. 0.88). The combined rs-FC and structural ML model showed the best performance (accuracy: 0.95, AUC: 0.95). Additionally, several rs-FC features in the final ML model correlated with FM’s clinical data. In conclusion, ML models based on rs-FC and brain structural MRI features could effectively differentiate FM patients from pain-free subjects.

## 1. Introduction

Fibromyalgia (FM) is a complicated chronic syndrome characterized by widespread pain, fatigue, sleep disorders, and psychological distress, considerably reducing patients’ quality of life [1]. The underlying pathomechanisms of FM have remained unclear, making diagnosis and treatment challenging [1,2,3]. Currently, FM is diagnosed based solely on clinical presentation, and the treatment for FM is just symptomatic therapy with limited efficacy in clinical practice [3,4]. 

Using electroencephalogram (EEG) and functional magnetic resonance imaging (MRI), studies observed that functional connectivity (FC) among several brain networks was impaired when patients performed tasks, were triggered by stimuli, or were during rest [5]. Specifically, several studies showed abnormal FCs in the triple networks, including the default mode network, salience network, and central executive network (frontoparietal network), considered the chronic pain pathway integrating physical and psychological domains [6,7,8,9]. Moreover, those abnormalities in these networks correlated with FM patients’ clinical presentations [6,7]. Moreover, altered FCs of the sensorimotor network have been shown to be associated with dysfunctional pain processing in FM [10]. 

The brain structural changes were also identified as the pathological features of FM [5]. Gray matter volume changes have been observed in several specific regions, such as the orbitofrontal cortex and anterior cingulate cortex, correlated with pain and psychological symptoms in FM patients [11,12]. Volume alterations in the gray matter might reflect the pathohistological process under FM and serve as specific signatures to detect FM from healthy and other conditions [13]. 

Machine learning (ML) effectively supports the diagnosis and prognosis of diseases based on different data, including neuroimaging data [14]. A previous study showed that ML based on multisensory-stimulated functional MRI patterns could distinguish FM patients from a pain-free population [15]. Another study demonstrated that ML using anatomical brain volume predicted FM patients with acceptable accuracy compared to self-reported clinical data [16]. Because the alterations in network-based rs-FC and brain structure are the characteristics of FM, we hypothesized that both network-based rs-FC and brain structural data could classify FM patients from healthy controls using the ML approach. However, no study compares or combines the predictability of resting-state FC (rs-FC) and structural data in distinguishing FM patients from healthy controls. Therefore, our study investigated the FM predictability of network rs-FC data and structural data. In addition, the study was also to evaluate the correlation between the predictive features of ML models and clinical presentation in FM patients.

## 2. Materials and Methods

### 2.1. Participants

Thirty healthy participants and twenty-six FM patients were enrolled in this study. All patients initially met the American College of Rheumatology 2016 criteria for the classification of fibromyalgia [17] at the time of diagnosis, which was confirmed by an experienced specialist at Taipei Medical University Hospital. The inclusion criteria of FM patients were as follows: (1) using stable medication dosages at least one month before enrolling in this study [18,19] and (2) being able to write the informed consent. In addition, both healthy participants and FM patients were excluded from this study if (1) participants had other types of pain or any history of head injuries, major neurological disorders, drug abuse, or malignant diseases; (2) participants were pregnant; and (3) participants had contraindications to MRI. The procedure of this cross-sectional study was approved by the Institutional Review Board-Taipei Medical University (N201812078, the approval day: 14 February 2019). Written consent was obtained from participants before they participated in the study. 

### 2.2. Clinical Assessment

The clinical presentation of each participant was re-evaluated using standardized questionnaires prior to MRI acquisitions. For sleep quality evaluation, the Pittsburgh Sleep Quality Index (PSQI), a self-reported questionnaire consisting of seven components with nineteen questions, was used [20]. The score for each item of the PSQI ranges from 0 (“no difficulty”) to 3 (“severe difficulty”), with a maximum score of 21 [20]. Recent evidence shows that PSQI has good internal consistency and test-retest reliability in FM patients (>0.8) [21]. 

For anxiety and depression measurement, Beck’s anxiety inventory (BAI) [22] and Beck’s depression inventory version II (BDI) [23] were used. BAI and BDI are the self-reported questionnaires with 21 items, which are easy to use and to interpret. The score of each item ranges from 0 (“not severe at all”) to 3 (“very severe”), and the maximum score is 63 [22,23]. The total BAI score is categorized into four groups of anxiety severity, including normal (0–9), mild (10–18), moderate (19–29), and severe anxiety group (>29) [22], which has been shown to have high internal consistency (>0.9) and test-retest reliability (>0.8) [24]. The total BDI score is also categorized into four groups of depression severity, including normal (0–13), mild (14–28), moderate (29–35), and severe depression group (>35). BDI-II has been shown to have good internal consistency and construct validity for depression measurement in patients with chronic pain [25].

For pain intensity, the Visual Analog Scale (VAS), which is a ten-centimeter ruler ranging from 0 (“no pain”) to 10 (“the worst imaginable pain”), was used to examine the patients at the clinical assessment time. To measure pain widespreadness and fibromyalgia impacts, the widespread pain index (WPI) was used to assess the pain widespreadness in FM patients by measuring painful areas in patients [17]. Recent evidence shows that WPI has an acceptable construct validity and reliability in chronic pain youth patients [26].

The levels of fibromyalgia symptoms in patients during the previous week were assessed using the Symptom Severity Scale (SSS) and Fibromyalgia Impact Questionnaire (FIQ). SSS mainly focuses on the severity of fatigue, cognitive symptoms, and unrefreshed waking up, with scores ranging from 0 (“no problem”) to 3 (“severe”) for each symptom and a maximal total score of 12 [17]. SSS has an acceptable reliability (0.7) [26]. FIQ, a ten-item questionnaire, is designed to measure the health status of FM patients, including physical functions, pain, fatigue, work status, stiffness, anxiety, and depression [27]. Internal consistency of FIQ is >0.8, whereas test-retest reliability of FIQ ranges from 0.56 to 0.95 [28].

To measure pressure pain threshold (PPT), the assessment sites were the nine paired tender points of the diagnostic criteria for FM defined by the ACR in 1990 [29], which are located at the occiput, low cervical, trapezius, supraspinatus, second rib, lateral epicondyle, gluteal, greater trochanter, and knee. During the measurement, the subject took a relaxed sitting position. Pressure pain thresholds were measured three times on each side and averaged.

### 2.3. MRI Acquisitions

All image data were acquired by a 3T MRI system (MAGNETOM Prisma; Siemens Healthcare, Erlangen, Germany) with a 20-channel head coil. During the MRI acquisition process, the participants were instructed to stay awake with closed eyes. T1-weighted high-resolution structural images were collected, using a 3D magnetization-prepared rapid gradient-echo sequence: repetition time (TR) = 2000 ms, echo time (TE) = 2.3 ms, flip angle = 8^0^, pixel matrix = 256 × 256, voxel-size = 1 mm × 1 mm × 1 mm, number of slices = 192, and slide order = interleaved. T2*-weighted functional images were obtained by a gradient echo sequence: TR = 2720 ms, TE = 24 ms, flip angle = 84^0^, pixel matrix = 64 × 64, voxel-size = 3 mm × 3 mm × 3 mm, number of slices = 50, and slide order = interleaved. All image data were recorded in DICOM format and then converted to NIFTI format.

### 2.4. Functional MRI Preprocessing and Resting-State Functional Connectivity Matrix Extraction

The CONN toolbox version 21a (The Gabrieli Lab, McGovern Institute for Brain Research, MIT) is based on Statistical Parametric Mapping software version 12 (SPM12; The Wellcome Department of Imaging Neuroscience, London) and implemented in MATLAB 2022a (The MathWorks Inc., Natick, MA, USA) was used to preprocess MRI data [30]. The imaging data were first realigned by co-registering and resampling all scans to a reference image. The SPM slice-timing correction procedure was applied to correct misalignment among slices of functional data, and then potential outliers were identified. Subsequently, the data were normalized into MNI space and were smoothed with a Gaussian kernel of 8 mm full width half maximum (FWHM). The anatomical component-based noise correction procedure was used to estimate and remove potential confounders from the estimated BOLD signal. The BOLD signal with temporal frequencies smaller than 0.008 or higher than 0.09Hz from the imaging data was removed using a band-pass filter.

After imaging preprocessing, the first level of ROI-to-ROI analysis was performed to extract the network-based rs-FC matrix. The ROIs were defined using the HCP network atlas built-in CONN toolbox [30], resulting from independent component analysis on the Human Connectome Project dataset. The atlas defined 32 nodes of 8 networks, including the default mode network, salience network, sensorimotor network, visual network, dorsal attention network, frontoparietal network, language network, and cerebellar network. For each subject, the time series of voxels in each ROI (node) were averaged, and the correlations between the averaged time series among ROIs were calculated. The ROI-to-ROI correlation coefficients were Fisher-transformed to z-scores for normalization. The z-score matrix was extracted for further analyses, including (32 × 31)/2 = 496 ROI-to-ROI z-scores for each subject. 

### 2.5. Voxel-Based Morphology Analyses and Gray Matter Volume Matrix Extraction

Voxel-based morphology (VBM) analyses were performed using the Computational Anatomy Toolbox (CAT12) in SPM12 [31]. In brief, the T1-weighted high-resolution structural images were segmented into gray matter, white matter, and cerebrospinal fluid. Subsequently, the images were normalized to MNI and smoothed with a Gaussian kernel of 8 mm FWHM. The gray matter volume matrix of 400 anatomical regions, defined by the 400-parcellation network atlas of Schaefer (2018) [32], was extracted for further analysis. 

### 2.6. Machine Learning Analysis

**Figure 1:**
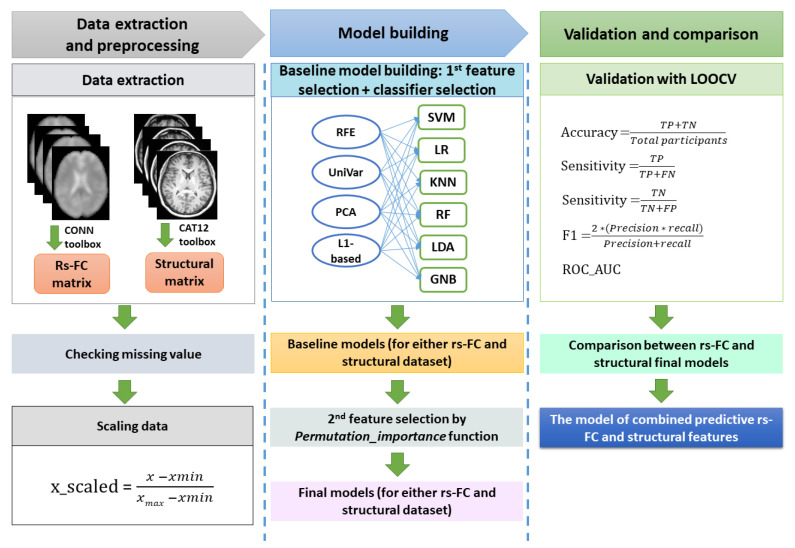
The machine learning procedure in this study. After extracting resting-state functional connectivity (rs-FC) data and structural data, both datasets were checked for missing values. Because rs-FC data were z-scores already, we just scaled structural data using the min-max scaling method. The baseline machine learning models for either the rs-FC dataset or structural dataset were built by selecting the best model among cross-combinations between four feature selection methods and six classifiers. Subsequently, feature importance function was applied on baseline models to remove features that had zero or negative features important scores, resulting in the final machine learning model for each of the rs-FC dataset and structural dataset. The performances of the two final models were compared to determine which was better for FM prediction. Finally, the predictive rs-FC and structural features were used to build the combined model to investigate the predictability of both functional and structural data. The validation for all models in this study were conducted with leave-one-out cross-validation (LOOCV). The performance matrix included accuracy, sensitivity, specificity, f1-score, and ROC_AUC. Note: TP = true positive; TN = true negative; FP = false positive; FN: false negative.

Our ML pipeline was divided into data processing, model building (baseline and optimal models), and validation (Figure 1). All steps were performed using Python 3.7 and the Scikit-learn 1.1.2 package [33].

#### 2.6.1. Preprocessing Data

For data processing, the datasets were checked for missing values. Then the structural dataset was scaled by the Min-Max scaling method (the functional data did not need to be scaled because those were z-score values already). 

#### 2.6.2. Feature Selection Methods for Selecting Baseline Models

It should be noted that both rs-FC and structural MRI data extracted from participants are high-dimensional, which includes noise and could induce overfitting for classification results [14]. Therefore, the feature selection was performed for those datasets. When building the baseline ML models in this study, several common feature selection methods were used, including recursive feature elimination (RFE), univariate feature selection, principal component analysis (PCA), and the L1-based selection method [33]. 

RFE is a wrapper method to select important features for classification or regression which is commonly used in ML studies with high-dimensional data [34,35]. Two main parameters needed to be set for the RFE method: the estimator and the number of selected features. In this study, logistic regression was applied as an estimator of RFE. The optimal number of features that best-distinguished FM patients from healthy controls was determined by performing the loop of RFE with leave-one-out cross-validation (RFECV function). The univariate feature selection method (“SelectKBest” technique) uses the univariate regression approach to find the most important features for classification [36]. In the current study, the F-test classification score was set as a score function, and the number of features that produced the best performance was chosen. PCA is the method to reduce the dimension of large data but still preserve the original data information [37]. The number of components was set to account for 95% of the variance of the input. The L1-based feature selection method is based on a linear model penalized with the l1 norm to remove the features having zero coefficients with the outcome [38]. This study used a linear support vector machine (LinearSVC) for feature selection with the parameter C set at 0.1.

#### 2.6.3. Classification Algorithms and Hyperparameter Optimization to Build Baseline Models

To find good performance baseline ML models, we used the cross-combination strategy in which each feature selection method mentioned above was combined with several classifiers [39,40]. We investigated six classification algorithms: support vector machine (SVM), Logistic Regression (LR), k-nearest neighbors (KNN), random forest (RF), linear discriminative analysis (LDA), and Gaussian Naïve Bayes (GBN), which were commonly used in machine learning of MRI data [41,42,43,44,45]. The hyperparameter optimization processes with the GridSearchCV method were conducted for all selection method + classifier combinations. The C, gamma, and kernel parameters were tuned for the SVM classifier. The C, penalty, and solver parameters were optimized for the LR classifier. The algorithm, number of neighbors, power, and weight parameters were adjusted in KNN classification. The criterion, max_features, and the number of estimator parameters for the RF classifier were tuned. The solver parameter was optimized for LDA classification, and the variance smoothing was adjusted for GNB classification. The combination of feature selection and classifier that showed the best performance among the others was selected as the baseline ML model for further building the final ML models. 

#### 2.6.4. Permutation Feature Importance Ranking to Building the Final Models for Each Data Type and the Combined Model

A permutation feature importance function was applied to detect and remove noise in the baseline ML models. The features that showed zero or negative permutation importance scores (usually observed in small datasets such as ours) were removed, resulting in the final ML model for each rs-FC dataset and structural dataset. The two final models were compared in all measurement indicators to determine the better classification datatype of FM. Finally, the ML model of combined FC and structural features was built to investigate the predictability of combined functional and structural features in distinguishing FM patients from healthy controls.

#### 2.6.5. Defining the Classification Performance Matrix

Due to the small sample size, the performance of feature selection methods and validation processes in this study was obtained by leave-one-out cross-validation, which has been suggested to reduce the risk of overfitting. Measurement indicators were recorded, including accuracy, sensitivity, specificity, f1, and ROC_AUC scores. In addition, the permutation test (repeated 1000 times) was performed to evaluate the classifying ability of each model. A permutation *p*-value < 0.05 was considered significant [46].

### 2.7. Correlation with Clinical Data

The data were expressed as mean ± standard deviation (continuous variables) or ratio (for gender). The Shapiro–Wilk test was used to evaluate the normal distributions of variables. The differences in demographic and clinical data were investigated by the Independent Sample *T*-test (for normally distributed variables) or Mann–Whitney U test (for non-normally distributed variables). A correlation analysis between all features of the final ML models and clinical measurement data (PSQI, BDI, BAI, WPI, FIQ, SSS, VAS, and PPT) was conducted using Kendall’s rank correlation method with false discovery rate (FDR) correction for multiple correlations. Statistical analyses were performed by R version 4.1.2 (R Foundation for Statistical Computing, Vienna, Austria) and Jeffreys’s Amazing Statistics Program (JASP) version 0.16.3 (The JASP Team; available at: https://jasp-stats.org/). A *p*-value < 0.05 was considered significant.

## 3. Results

### 3.1. Demographic and Clinical Characteristics

Age, gender, and BMI had no significant difference between the two groups. However, the PSQI, BAI, and BDI scores in the FM group were significantly higher than those in the control group (*p* < 0.05). Pain intensity (VAS score), pain widespreadness (WPI score), and FM impacts (FIQ and SSS scores) were recorded in the FM group but not detected in the control group (Table 1).

### 3.2. Fibromyalgia Classification Using Resting-State Functional Connectivity Data

#### 3.2.1. Comparison of Cross-Combination Models and Baseline rs-FC ML Model Selection

The numbers of rs-FC features (or components for PCA) selected by RFE, univariate, PCA, and L1-based methods were 14, 11, 44, and 2, respectively. The RFE method outperformed the other feature selection methods, expressing high accuracy and AUC when combined with all classifiers. Regarding classifiers, after hyperparameter optimization, SVM and LR exhibited high accuracy compared with KNN, RF, LDA, and GNB classifiers combined with all feature selection methods (See Table S1 in the Supplementary Materials). Feature selection RFE + classifier SVM showed the highest accuracy (accuracy: 0.89, AUC = 0.93, *p* = 9.9 × 10^−4^), followed by RFE + classifier LR (accuracy: 0.88, AUC = 0.94, *p* = 9.9 × 10^−4^) and RFE + classifier LDA (accuracy: 0.84, AUC = 0.93, *p* = 9.9 × 10^−4^). In addition, RFE + classifier SVM also ranked first in sensitivity (0.85), specificity (0.93), and f1-score (0.88) in the rs-FC dataset. Thus, the SVM classifier with fourteen features selected by the RFE method was chosen as our baseline ML model of rs-FC data for further analyses (Table 2).

#### 3.2.2. The Final ML Model for rs-FC Data

**Figure 2:**
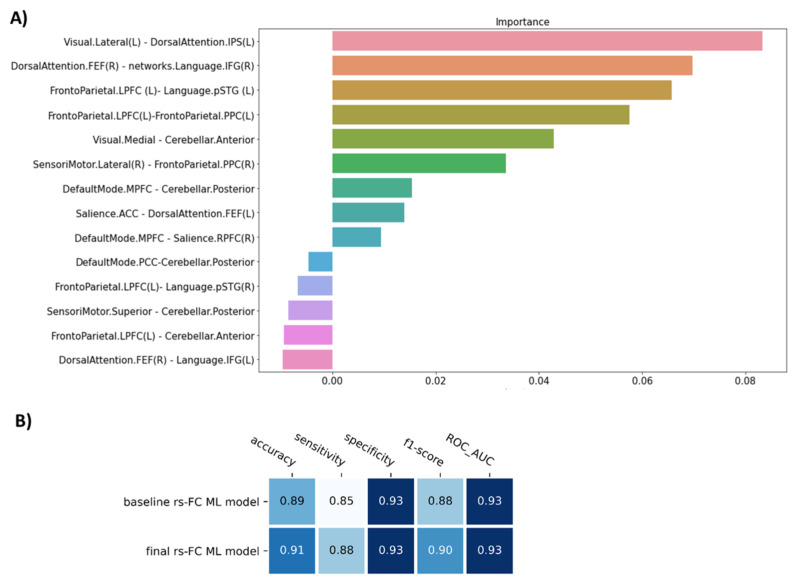
The figure show (**A**) The important feature ranking in the baseline model of resting-state functional connectivity data and (**B**) The performance of the model before (baseline rs-FC ML model) and after removing non-informative features (final rs-FC ML model). Note: Visual.Lateral (L) and Visual.Medial = the left lateral visual network and the medial visual network; DorsalAttention.IPS (L) and DorsalAttention.FEF (R) = the left intraparietal sulcus node and frontal eye field node of dorsal attention network; Language.IFG and Language.pSTG = the inferior frontal cortex node and the posterior superior temporal gyrus node of language network; Frontoparietal.LPFC and Frontoparietal.PPC = the lateral prefrontal cortex node and the posterior parietal cortex node of frontoparietal network; Cerebellar.Anterior and Cerebellar.Posterior = anterior and posterior node of cerebellum; SensoriMotor.Lateral and SensoriMotor.Superior = the lateral sensorimotor network and the superior sensorimotor network; DefaultMode.MPFC and DefaultMode.PCC = the medial prefrontal cortex node and posterior cingulate cortex node of the default mode network; Salience.ACC and Salience.RPFC = the anterior cingulate cortex node and the rostral prefrontal cortex node of the Salience network.

The permutation importance function was performed to rank the importance of 14 selected features in the baseline ML model. Five of fourteen features showed negative scores and thus were removed (Figure 2A). Therefore, the SVM model with nine features was applied as our final model of rs-FC data to classify FM patients from healthy controls. Compared to the baseline model, the final model was higher in accuracy (0.91), sensitivity (0.88), and f1-score (0.90), but similar in specificity (0.93) and AUC (0.93) (*p* = 9.9 × 10^−4^) (Table 2, Figure 2B).

### 3.3. Fibromyalgia Classification Using Structural Data

#### 3.3.1. Comparison of Cross-Combination Models and Selecting Baseline ML Model for Structural Data

The numbers of gray matter volume features (or components for PCA) selected by RFE, univariate, PCA, and L1-based methods were 9, 96, 44, and 1, respectively. RFE was also ranked as the best feature selection method in the structural dataset. Compared with the other classifiers, SVM and LR demonstrated good performances when combined with all feature selection methods (See Table S2 in the Supplementary Materials). RFE + classifier LR showed the highest accuracy (accuracy = 0.86, AUC = 0.86, *p* = 9.9 × 10^−4^), followed by RFE + classifier SVM (accuracy = 0.82, AUC = 0.89, *p* = 9.9 × 10^−4^) and RFE + classifier LDA (accuracy = 0.82, AUC = 0.89, *p* = 9.9 × 10^−4^). In addition, RFE + classifier LR also showed the highest sensitivity (0.85), specificity (0.87), and f1-score (0.85) in the structural dataset. Therefore, the LR classifier with nine features selected from the RFE method was chosen as our baseline ML model of structural data for further analyses (Table 3).

#### 3.3.2. The Final ML Model for Structural Data

**Figure 3:**
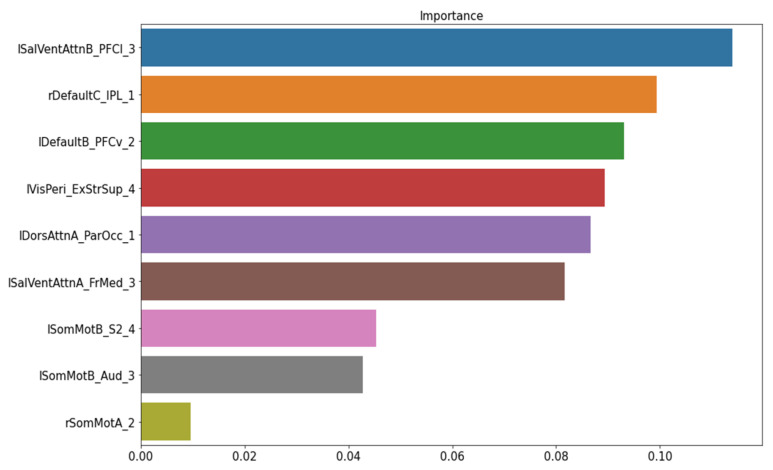
The important feature ranking in the baseline model of structural data. Note: lSalVentAttnB_PFCI_3 = the lateral prefrontal cortex regions of the left salience ventral attention network B; rDefaultC_IPL_1 = the right inferior parietal lobule node 1 of the default mode network C; lDefaultB_PFCv_2 = the ventral prefrontal cortex node 2 of the left default mode network; MisPeri_ExStrSup_4 = extra-striate superior node 4; lDorsAttnA_ParOcc_1 = parietal occipital node 1 of the left dorsal attention network A; lSalVentAttnA_FrMed_3 = the medial frontal node of the left salience ventral attention network A; lSomMotB_S2_4 = the S2 node 4 of the left sensorimotor network B; lSomMotB_Aud_3 = the auditory node 3 of sensorimotor network B; rSomMotA_2 = the right sensorimotor network A node 2.

The permutation importance function was performed to rank the importance of nine structural features in the baseline ML model. No feature showed a zero or negative score (Figure 3). Thus, the baseline model was kept as our final model of structural data to classify FM patients from healthy controls.

### 3.4. Comparison in the Classification Ability between Functional MRI Data and Structural Data

**Figure 4:**
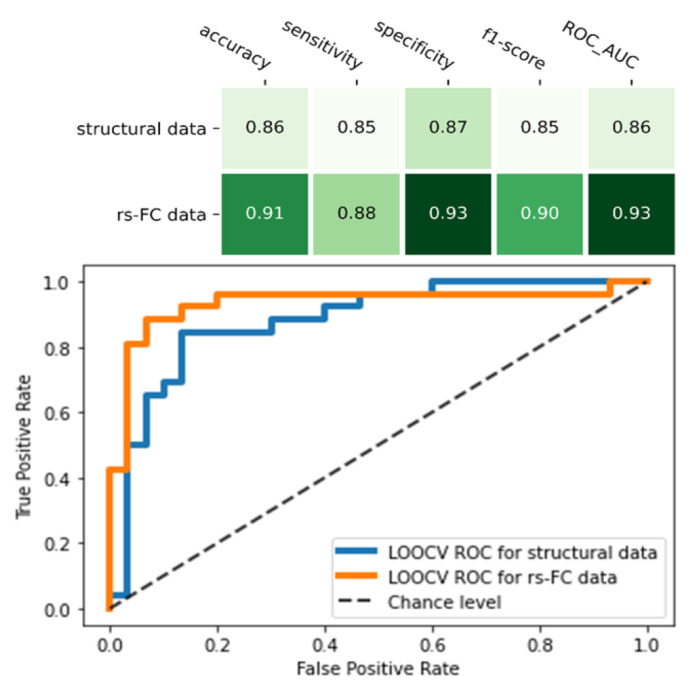
Comparison between two final machine learning models of resting-state functional connectivity data and structural data. The figure showed the performance matrix (the upper table) and the receiver operating characteristic (ROC) curve and its area under curve (AUC) score of the two models (the lower chart).

Compared to the final model of structural MRI data, the rs-FC ML model was higher in all indicators: accuracy (0.91 vs. 0.86), sensitivity (0.88 vs. 0.85), specificity (0.93 vs. 0.87), f1-score (0.91 vs. 0.85), and ROC_AUC score (0.93 vs. 0.88) (Figure 4). Of note, the rs-FC ML model also outperformed the structural model that used the same feature selection (RFE) and classifier (SVM) method (Table 3, Figure 4). 

### 3.5. Fibromyalgia Classification Using the Combination of Functional and Structural Data

We performed the ML model using both predictive FC and structural features from the final ML models of FC and structural data (Table S3). The combined model with SVM or LR showed the same performance, with accuracy: 0.95, sensitivity: 0.96, specificity: 0.93, f1-score: 0.95, and ROC_AUC score: 0.95 (*p*-value = 9.9 × 10^−4^). Compared to the final ML model of rs-FC features only, the model of combined functional and structural features showed higher in all indicators, except specificity. 

### 3.6. Correlation between the Selected Features in the Final ML Models with Clinical Data

**Figure 5:**
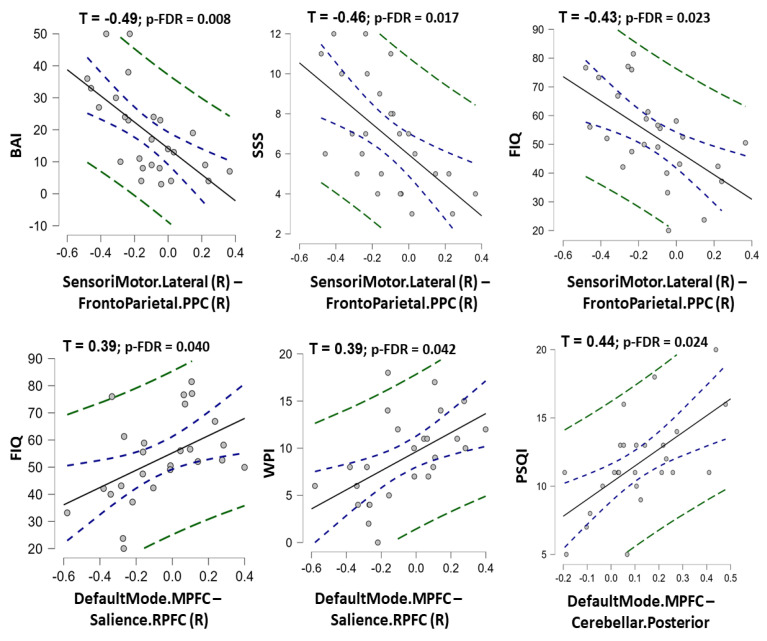
The charts show correlation between the predictive resting-state functional connectivity features and clinical measurement scores in fibromyalgia patients. The Kendall’s Tau correlation coefficients (with *p*-value corrected by false discovery rate) were shown for statistically significant correlations. Note: SensoriMotor.Lateral (R)–FrontoParietal.PPC (R) = the functional connectivity between the lateral sensorimotor network and the posterior parietal cortex node of frontoparietal network; DefaultMode.MPFC-Salience.RPFC (R) = the functional connectivity between the medial prefrontal cortex node of the default mode network and the rostral prefrontal cortex node of the Salience network; DefaultMode.MPFC-Cerebellar. Posterior = the functional connectivity between the medial prefrontal cortex node of the default mode network and the posteior part of cerebellum; PSQI = Pittsburgh Sleep Quality Index; BAI = Beck’s anxiety inventory; SSS = Symptom Severity Scale; FIQ = Fibromyalgia Impact Questionnaire; WPI = Widespread Pain Index.

Among nine rs-FC features in the final ML model of rs-FC data, the rs-FC between the right sensorimotor network and prefrontal parietal cortex was negatively correlated with BAI, SSS, and FIQ scores (τ = −0.49, −0.46, and −0.43 with p-FDR = 0.008, 0.017, and 0.023, respectively). In contrast, the rs-FC between the medial prefrontal cortex (default mode network) and the right rostral prefrontal cortex (salience network) was positively correlated with FIQ and WPI scores (Kendall’s Tau τ = 0.39 and 0.39 with p-FDR = 0.040 and 0.042, respectively). The rs-FC between the medial prefrontal cortex (default mode network) and the posterior cerebellar region (cerebellar network) was positively correlated with the PSQI score (τ = 0.44; p-FDR = 0.024) (Figure 5, Table S4). A significant correlation between structural features and clinical data was not found in this study (Table S5).

## 4. Discussion

Many studies have reported the alterations of network rs-FCs and brain structure in FM patients [7,47,48]. These alternations are considered to reflect the pathophysiology of FM [13,49]. However, the features extracted from structural and functional brain mapping are complex, making the interpretation difficult. Thus, our study used the ML approach to analyze the high dimensional data from the brain MRI. We successfully selected signatures of the neuroimaging data in FM. Using the SVM/LR classifier with two-step feature selection by RFE and permutation importance function, we found that both rs-FC and structural data could classify FM patients from healthy controls with high performance. The classification performance of rs-FC data was higher than that of structural data, as evidenced by the higher in all indicators, including accuracy, sensitivity, specificity, f1-score, and ROC-AUC. When combining the predictive rs-FC and structural features, our ML model showed the best performance compared to the models of each kind of FC or structural data only. In addition, the predictive rs-FCs of the final ML model were also significantly correlated with clinical measurement scores (PSQI, BAI, WPI, SSS, and FIQ). Our study suggested the predictive validity of rs-FC data and structural MRI data in FM. 

The cross-combination strategy could filter down irrelevant features and select the optimal ML model for classification [39,40]. Regarding feature selection methods, our study showed that the RFE method exhibited high performance when combined with all classifiers in building baseline ML models. In contrast, the PCA method expressed lower performance in most classifiers. Of note, RFE is the wrapper method that selects features based on predictive accuracy and has been widely used in previous ML studies of MRI data to achieve high performance [50,51,52]. Because PCA is the unsupervised technique for feature selection, this method might not always enhance the model performance in supervised ML [14,41]. After building the baseline models for both rs-FC and structural data, we applied the permutation importance function to further remove non-informative features. This feature selection method can calculate feature importance independent of the classifiers used and, thus, can be applied to most classifiers, even without native feature importance scores [46]. Regarding classifiers, the SVM or LR classifiers expressed high performances compared with the other classifiers across different feature selection methods in our study, which might be because those classifiers’ regularization could reduce the effect of noise in the dataset [14,41]. However, evidence also suggests that SVM or LR does not provide good performance in some cases of noisy MRI data, and thereby the dataset needs to undergo feature selection before applying SVM or LR training to reach the optimal performance [53]. The other classifiers, including GNB, LDA, and KNN, showed inferior performance compared with SVM or LR in our study, possibly because those classifiers are more sensitive to noise [41,53]. In sum, our study showed that SVM or LR with two steps of feature selection (RFE + permutation importance feature selection) might be one of the good options for ML studies using functional and structural MRI data to understand FM. 

Our study showed that the ML model of network-based rs-FC data using the SVM classifier with RFE + permutation importance feature selection could classify FM patients from healthy controls with high performance. In support of this, in a previous ML study analyzing the altered patterns of functional MRI after painful and non-painful stimulation in FM, the combined activity of altered patterns effectively predicted the FM from healthy control, whose accuracy, sensitivity, and specificity were 93%, 92%, and 94%, respectively [15]. The altered patterns in this study [15] involved several anatomical regions of the salience network (i.e., insula, operculum, and anterior cingulate cortex), the default mode network (i.e., medial frontal cortex and posterior cingulate cortex), and the sensorimotor network (i.e., sensory cortices), which were consistent with our findings. Another study evaluated the predictability of rs-FC MRI data on chronic pain, showing that deep learning with the Ann4brain architecture model classified FM and chronic back pain patients from non-pain participants with 86.8% accuracy and 91.8% ROC_AUC, in which the predictive features were also rs-FC among the default mode network, salience network, frontoparietal network, and cerebellum [54]. Additionally, a previous study showed that the ML model of rs-FC data could classify patients with migraine (without aura) from healthy controls and other chronic pain disorders such as FM, also based on rs-FC among the networks mentioned above [55]. Altogether, accumulative evidence suggests that rs-FC data might be used to classify chronic pain patients such as FM from pain-free controls. The predictive FC features mainly belong to the triple networks and the sensorimotor network, repeatedly suggesting the important role of those networks in chronic pain disorders, including FM. However, each kind of chronic pain may have its own specific predictive patterns of rs-FC. 

In addition to rs-FC data, our study found that the ML model of network-based gray matter volume could also distinguish FM patients from healthy controls. The best model using the LR classifier with RFE + permutation importance feature selection achieved a high accuracy of 85%. A previous study used the J48-decision tree classifier on anatomical gray volume to predict FM patients, reaching 76% accuracy [16]. In this study [16], the altered volumes of the cerebellum, cerebral cortex, and basal ganglia were used to predict FM. In contrast, our study using a network-based atlas showed that the brain regions belonging to the triple networks and sensorimotor network were predictive features compatible with our results in rs-FC data. Differences in performance between our study and the mentioned study [16] might be partially due to different atlases (anatomical vs. network-based atlases), ages, and severe stages of FM patients in the two studies. In addition, evidence suggests that the brain structure in FM is specifically changed by age, making the brain structure alterations in FM vary across the samples [56]. The global volume of the brain was also unchanged in previous reports [56]. However, both studies suggested that the ML model using structural MRI features could outperform the chance to predict FM patients. Our study also emphasized that the structural alterations of triple and sensorimotor networks could be the critical pathological characteristics in FM, in addition to those networks’ rs-FC. 

To the best of our knowledge, our study was the first to compare the ML performance between rs-FC and structural features in FM classification. We found that the rs-FC model outperformed the structural model in all indicators. Although both data types can be used for FM classification, the finding implied that rs-FC data might be preferred to structural data in the ML approach. It should be noted that FM interacts with age to change brain structure, making the changes in gray matter volume not directly correlated with FM status [56]. Evidence suggests that long-term FM duration might be needed to re-organize brain structure [57]. Furthermore, gray matter volume variation in FM might be not only based on pain symptoms but also cognitive and psychological symptoms that are affected by several aspects [58]. In addition, abnormal neural plasticity and neuroinflammation could affect the volumes of specific brain regions, leading to variations in structural changes in FM patients [59]. Therefore, rs-FC data might be more sensitive and specific for FM prediction than structure data. 

The study showed that the model of combined rs-FC and structural data outperformed the ML model of rs-FC data only. Consistently, a previous ML study using functional and structural MRI features discriminated migraine patients (without aura) from healthy controls with 83.67% accuracy, 92.86% sensitivity, and 71.43% specificity [45]. Another study using multimodal neuroimaging and autonomic signals to predict clinical pain in patients with chronic low back pain, achieving 92.45% accuracy and 97% ROC_AUC [60]. The findings might imply that the combined different feature types could be superior to the single feature type in distinguishing among health conditions. 

Moreover, we found that the predictive rs-FCs were related to both pain and psychological symptoms, supported by correlations between the selected features of our ML model and clinical measurement scores (PSQI, BAI, WPI, FIQ, and SSS). Those features mainly belonged to the triple and sensorimotor networks, which have been hypothesized to be involved in pain processing in FM [9,10]. Specifically, the rs-FC between the right lateral sensorimotor network and the posterior parietal cortex of the frontal-parietal network was negatively correlated to BAI, FIQ, and SSS scores, meaning that the more hypo-connectivity in FC between these networks might reflect the higher level of anxiety and FM impacts, which is consistent with previous studies showing that dysfunction in this network might induce anxiety disorders and pain dysregulation in FM [10,61]. In addition, our study found that rs-FC between the default mode network and salience network, as well as rs-FC between the default mode network and cerebellum, were positively correlated with WPI and FIQ, meaning that the stronger FC between the two networks might worsen the FM presentation in patients. Consistently, a previous study showed that FC between the default mode network and salience network was positively correlated with pain widespreadness and pain catastrophizing in FM [19]. In addition, FC alterations in the default and salience networks were repeatedly observed and found to be significantly related to FM symptoms [6,8,62]. The previous study showed that clinical pain was also correlated with the altered patterns of multisensory-response functional MRI features in the triple networks and the sensorimotor network [15]. In sum, the selected features of the final rs-FC model could help predict FM from healthy controls. Because those features were related to presentation in FM, our findings might suggest further investigation regarding the therapeutic methods for FM that modulate the FC among triple and sensorimotor networks to reduce pain and psychological symptoms [9]. The application of noninvasive brain stimulation (such as noninvasive transcranial electrical stimulation) in neural networks integrated with predictive artificial intelligence in chronic pain has been introduced. It is promised to reduce medication usage in patients, but evidence for its efficacy is still lacking [63]. Moreover, a study found that the alterations of migraine headache frequency caused by treatment correlated with FC alteration [55], which might imply that FCs could predict treatment responses in FM. 

There were several limitations of the current study which need to be concerned. First, we did not carry out external validation due to the small sample size. However, we applied the integration of leave-one-out cross-validation and permutation test to evaluate the ML models, which could reduce the risk of overfitting. Second, we did not have the group of other chronic pain and disorders that might be misdiagnosed with FM. Our model could classify FM patients from healthy controls, but we did not know whether rs-FC and structural data could classify FM from those diseases. Third, this study did not apply deep learning classification due to sample size limitations. Our study was cross-sectional, which cannot evaluate the temporal alterations of rs-FC and predictive features in FM. All of those limitations encourage us to recruit a larger sample size of FM patients and other disorders, such as chronic fatigue syndrome, rheumatoid arthritis, osteoarthritis, or depression, to investigate the predictability of the ML model to classify FM from other disorders, thereafter compare the performance of the deep learning model to other ML models, as well as investigate the time-based alterations of the predictive ML model in FM. Finally, the study population is not treatment naïve for FM. Therefore, the potential effects of treatments (e.g., medications) cannot be excluded from the present study.

## 5. Conclusions

In conclusion, our study found that both rs-FC and structural MRI data could classify FM patients from healthy controls, but the former outperformed the latter. The ML model of combined functional and structural data showed the best performance in FM classification. The predictive rs-FC features correlated with clinical presentation in FM. Those FCs mainly belong to the triple networks and the sensorimotor network, repeatedly supporting the hypothesis that those networks might involve in chronic pain processing, including FM. The current study also showed that the combination of functional and structural features was superior to a single kind of data type in distinguishing FM from healthy controls. Because FCs correlate with clinical presentation, we might further hypothesize that MRI features could predict treatment responses to therapeutic modalities in FM. Future studies might be conducted to evaluate the alterations of network FCs in response to the improvement of different therapeutic methods, such as transcranial direct current stimulation or repetitive transcranial magnetic stimulation, to further clarify the predictability of MRI features in the treatment of FM. Moreover, it should be noted that, in addition to the triple networks and the sensorimotor network, FCs and structural features of the visual network, emerging as a contributor to pain processing [64], were a predictive feature of our ML model. Both FC and structural MRI alterations of the visual networks in FM remain unclear and in need of further investigation. 

## Figures and Tables

**Table 1:** Demographic and clinical characteristics.

	FM (*n* = 26)	Control (*n* = 30)	*p*-Value
Age	49.6 ± 11	52.1 ± 10.5	0.393
Gender (Female/male)	25/1	28/2	1.000
BMI (kg/m^2^)	21.6 ± 3.8	23.2 ± 4.5	0.183
PSQI	11.7 ± 3.5	5.3 ± 2.4	2.1 × 10^−8^
BAI	19.2 ± 13.8	4.1 ± 4.2	5.5 × 10^−7^
BDI	18.7 ± 12.9	4.6 ± 4.5	4.3 × 10^−7^
VAS	5.6 ± 2.3	-	-
WPI	8.9 ± 4.5	-	-
SSS	6.9 ± 2.8	-	-
FIQ	53.1 ± 16.0	-	-
PPT (kg/cm^2^)	2.37 ± 1.14	-	-

The table shows the demographic characteristics as well as clinical presentation of fibromyalgia patients (FM) and the healthy controls measured by Pittsburgh Sleep Quality Index (PSQI), Beck’s anxiety inventory (BAI), Beck’s depression inventory version II (BDI), Visual Analog Scale (VAS), Widespread Pain Index (WPI), Symptom Severity Scale (SSS), Fibromyalgia Impact Questionnaire (FIQ), and Pain Pressure Threshold (PPT). The results are expressed as mean ± standard deviation (except for the gender variable expressed as the number of females to males). The differences between the two groups were examined by Independent Sample *T*-test (for Age and BMI), the Mann–Whitney U test (for PSQI, BAI, and BDI), and Chi-square test (for Gender).

**Table 2:** Comparing performance among different combinations of four feature selection methods and six classifiers in resting-state functional connectivity dataset.

Selection Method	Classifier	Accuracy	Sensitivity	Specificity	F1-Score	ROC_AUC	*p*-Value (Permutation Test)
RFE (14 features)	SVM	0.89	0.85	0.93	0.88	0.93	9.9 × 10^−4^
	LR	0.88	0.85	0.90	0.86	0.94	9.9 × 10^−4^
	KNN	0.79	0.81	0.77	0.78	0.83	9.9 × 10^−4^
	RF	0.77	0.81	0.73	0.76	0.83	9.9 × 10^−4^
	LDA	0.84	0.85	0.83	0.83	0.93	9.9 × 10^−4^
	GNB	0.79	0.81	0.77	0.78	0.89	9.9 × 10^−4^
Univar (11 features)	SVM	0.77	0.69	0.83	0.73	0.80	9.9 × 10^−4^
	LR	0.82	0.77	0.87	0.80	0.87	9.9 × 10^−4^
	KNN	0.73	0.73	0.73	0.72	0.80	0.002
	RF	0.71	0.69	0.73	0.69	0.74	0.002
	LDA	0.73	0.69	0.77	0.71	0.82	9.9 × 10^−4^
	GNB	0.75	0.77	0.73	0.74	0.85	9.9 × 10^−4^
PCA (44 components)	SVM	0.59	0.54	0.63	0.55	0.17	0.175
	LR	0.55	0.54	0.57	0.53	0.56	0.283
	KNN	0.61	0.65	0.57	0.61	0.61	0.089
	RF	0.55	0.46	0.63	0.49	0.55	0.266
	LDA	0.45	0.42	0.47	0.42	0.45	0.753
	GNB	0.39	0.31	0.47	0.32	0.32	0.889
L1-based (2 features)	SVM	0.68	0.85	0.53	0.71	0.63	0.008
	LR	0.66	0.65	0.67	0.64	0.68	0.013
	KNN	0.61	0.58	0.63	0.58	0.61	0.110
	RF	0.64	0.62	0.67	0.62	0.64	0.027
	LDA	0.64	0.65	0.63	0.63	0.67	0.024
	GNB	0.66	0.69	0.63	0.65	0.69	0.018

The table shows a performance matrix of different combinations of selection methods and classifiers using resting-state functional connectivity data. The feature selection methods include recursive feature elimination (RFE), univariate feature selection (Univar), principal component analysis (PCA), and L1-based feature selection method (L1-based). The classifiers include support vector machine (SVM), logistic regression (LR), k-nearest neighbors (KNN), random forest (RF), linear discriminative analysis (LDA), and Gaussian Naïve Bayes (GNB). Permutation test was used to test the significance of each model, which a significant level was 0.05.

**Table 3:** Comparing performance among different combinations of four feature selection methods and six classifiers in structural data.

Selection Method	Classifier	Accuracy	Sensitivity	Specificity	F1-Score	ROC_AUC	*p*-Value (Permutation Test)
RFE (9 features)	SVM	0.82	0.85	0.80	0.81	0.89	9.9 × 10^−4^
	LR	0.86	0.85	0.87	0.85	0.86	9.9 × 10^−4^
	KNN	0.79	0.85	0.73	0.79	0.79	0.002
	RF	0.71	0.73	0.70	0.70	0.64	0.217
	LDA	0.82	0.81	0.83	0.81	0.89	9.9 × 10^−4^
	GNB	0.70	0.65	0.73	0.67	0.78	0.004
Univar (96 features)	SVM	0.71	0.73	0.70	0.70	0.76	0.006
	LR	0.77	0.73	0.80	0.75	0.76	9.9 × 10^−4^
	KNN	0.63	0.81	0.47	0.67	0.60	0.415
	RF	0.66	0.62	0.70	0.63	0.58	9.9 × 10^−4^
	LDA	0.57	0.73	0.43	0.61	0.66	0.225
	GNB	0.55	0.58	0.53	0.55	0.62	0.327
PCA (44 components)	SVM	0.59	0.54	0.63	0.55	0.24	0.146
	LR	0.55	0.54	0.57	0.53	0.59	0.285
	KNN	0.50	0.85	0.20	0.61	0.44	0.408
	RF	0.53	0.54	0.53	0.52	0.53	0.250
	LDA	0.38	0.42	0.33	0.39	0.40	0.948
	GNB	0.46	0.42	0.50	0.42	0.46	0.644
L1-based (1 feature)	SVM	0.70	0.62	0.77	0.65	0.55	0.007
	LR	0.70	0.62	0.67	0.65	0.64	9.9 × 10^−4^
	KNN	0.61	0.73	0.50	0.63	0.58	0.096
	RF	0.48	0.38	0.57	0.41	0.46	0.618
	LDA	0.70	0.62	0.77	0.65	0.64	0.002
	GNB	0.61	0.50	0.70	0.54	0.59	0.071

The table shows a performance matrix of different combinations of feature selection methods and classifiers, using structural MRI data. The feature selection methods include recursive feature elimination (RFE), univariate feature selection (Univar), principal component analysis (PCA), and L1-based feature selection method (L1-based). The classifiers include support machine vector (SVM), logistic regression (LR), k-nearest neighbors (KNN), random forest (RF), linear discriminative analysis (LDA), and Gaussian Naïve Bayes (GNB). Permutation *p*-value was used to test the significance of each model, which a significant level was 0.05.

## Data Availability

The datasets generated and/or analyzed during the current study are available from the corresponding author on reasonable request.

## Supplementary Materials

The following supporting information can be downloaded at https://www.mdpi.com/article/10.3390/biomedicines10123002/s1, Table S1: The optimal parameters selected for each cross-combination between four feature selection and six classification methods in resting-state functional connectivity data; Table S2: The optimal parameters selected for each cross-combination between four feature selection and six classification methods in structural data; Table S3. Performance of the combined ML model of both functional and structural features; Table S4: Correlation coefficients between predictive rs-FC features and clinical data; Table S5: Correlation coefficients between predictive structural features and clinical data.
