# Supplementary material for: Identification of Resting-State Network Functional Connectivity and Brain Structural Signatures in Fibromyalgia Using a Machine Learning Approach
Source: Biomedicines. 2022 Nov 22;10(12):3002. doi: 10.3390/biomedicines10123002 (PMC9775534; doi:10.3390/biomedicines10123002)
Supplement: Supplementary file 1 [file biomedicines-10-03002-s001.zip › biomedicines-1995377-supplementary.pdf]

biomedicines

MDPI

Supplement materials

Table S1. The optimal parameters for each cross-combination between four feature selection and six classification methods in resting-state functional connectivity data

Selection Method	Classifier	Parameters
RFE	SVM	C = 100, gamma = ‘auto’, kernel = ‘rbf’
	LR	C = 10, penalty = ‘l2’, solver = ‘saga’
	KNN	algorithm = 'auto', n_neighbors = 3, p = 3, weights = 'uniform'
	RF	criterion = 'gini', max_features = 'sqrt', n_estimators = 100
	LDA	solver = 'svd'
	GNB	var_smoothing = 1e-11
Univar	SVM	C = 1, gamma = 'scale', kernel = 'rbf'
	LR	C = 1, penalty = 'l2', solver = 'saga'
	KNN	algorithm = 'auto', n_neighbors = 5, p = 2, weights = 'uniform'
	RF	criterion = 'gini', max_features = 'sqrt', n_estimators = 10
	LDA	solver = 'svd'
	GNB	var_smoothing = 1e-11
PCA	SVM	C = 10, gamma = 'scale', kernel = 'sigmoid'
	LR	C = 1, penalty = 'l1', solver = 'saga'
	KNN	algorithm = 'auto', n_neighbors = 1, p = 1, weights = 'uniform'
	RF	criterion = 'gini', max_features = 'sqrt', n_estimators = 1
	LDA	solver = 'svd'
	GNB	var_smoothing = 1e-11
L1-based	SVM	C = 0.1, gamma = 'scale', kernel = 'poly'
	LR	C = 10, penalty = 'l2', solver = 'saga'
	KNN	algorithm = 'auto', n_neighbors = 3, p = 1, weights = 'distance'
	RF	criterion = 'gini', max_features = 'sqrt', n_estimators = 1
	LDA	solver = 'svd'
	GNB	var_smoothing = 1e-11

Table S2. The optimal parameters for each cross-combination between four feature selection and six classification methods in structural data

Selection Method	Classifier	Parameters
RFE	SVM	C = 10, gamma = 'scale', kernel = 'linear'
	LR	C = 10, penalty = 'l1', solver = 'liblinear'
	KNN	algorithm = 'auto', n_neighbors = 5, p= 1, weights = 'uniform'
	RF	criterion = 'gini', max_features = 'sqrt', n_estimators = 10
	LDA	solver = 'svd'
	GNB	var_smoothing = 1e-11
Univar	SVM	C = 10, gamma = 'auto', kernel = 'rbf'
	LR	C = 1, penalty = 'l2', solver = 'liblinear'
	KNN	algorithm = 'auto', n_neighbors = 2, p = 3, weights = 'uniform'
	RF	criterion = 'gini', max_features = 'sqrt', n_estimators = 100
	LDA	solver = 'svd'
	GNB	var_smoothing = 1e-11
PCA	SVM	C = 10, gamma = 'scale', kernel = 'sigmoid'
	LR	C = 1, penalty = 'l1', solver = 'saga'
	KNN	algorithm = 'auto', n_neighbors =2, p = 2, weights = 'uniform'
	RF	criterion = 'gini', max_features = 'log2', n_estimators = 10
	LDA	solver = 'svd'
	GNB	var_smoothing = 1e-11
L1-based	SVM	C = 10, gamma = 'auto', kernel = 'sigmoid'
	LR	C = 10, penalty = 'l1', solver = 'liblinear'
	KNN	algorithm = 'auto', n_neighbors = 4, p = 1, weights = 'uniform'
	RF	criterion = 'gini', max_features = 'sqrt', n_estimators = 1
	LDA	solver = 'svd'
	GNB	var_smoothing = 1e-11

Table S3. Performance of the combined ML model of both functional and structural features

Classifier	Parameters	Accuracy	Sensitivity	Specificity	F1-score	ROC_AUC
SVM	C = 10, gamma = 'scale', kernel = 'linear'	0.95	0.96	0.93	0.95	0.95
LR	C = 10, penalty = 'l1', solver = 'liblinear'	0.95	0.96	0.93	0.95	0.95
KNN	algorithm = 'auto', n_neighbors = 5, p= 1, weights = 'uniform'	0.84	0.81	0.87	0.85	0.84
RF	criterion = 'gini', max_features = 'sqrt', n_estimators = 10	0.69	0.90	0.89	0.93	0.80
LDA	solver = 'svd'	0.91	0.92	0.90	0.92	0.91
GNB	var_smoothing = 1e-11	0.86	0.88	0.83	0.86	0.78

The table shows a performance matrix of different combinations of feature selection methods and classifiers, using structural MRI data. The feature selection methods include recursive feature elimination (RFE), univariate feature selection (Univar), principal component analysis (PCA), and L1-based feature selection method (L1-based). The classifiers include support machine vector (SVM), logistic regression (LR), k-nearest neighbors (KNN), random forest (RF), linear discriminative analysis (LDA), and Gaussian Naïve Bayes (GNB). Permutation p-value of all those models are 9.9e-4.

Table S4. Correlation coefficients between predictive rs-FC features and clinical data

Variable	PSQI	BAI	BDI	WPI	SSS	FIQ	VAS	PPT
DefaultMode.MPFC_Salience.RPFC(R)	0.26	0.23	0.26	**0.39***	0.21	**0.39***	-0.02	-0.04
	(0.262)	(0.340)	(0.262)	**(0.042)**	(0.382)	**(0.040)**	(0.929)	(0.871)
DefaultMode.MPFC_Cerebellar.Posterior	**0.44***	0.15	0.18	0.28	0.23	0.13	0.10	-0.08
	**(0.024)**	(0.532)	(0.463)	(0.205)	(0.348)	(0.598)	(0.671)	(0.728)
SensoriMotor.Lateral(R)_FrontoParietal.PPC(R)	-0.32	**-0.49***	-0.29	-0.20	**-0.46***	**-0.43***	-0.17	0.06
	(0.141)	**(0.008)**	(0.163)	(0.425)	**(0.017)**	**(0.023)**	(0.490)	(0.785)
Visual.Medial_Cerebellar.Anterior	-0.02	-0.07	0.11	0.19	-0.15	-0.11	0.05	-0.06
	(0.931)	(0.785)	(0.626)	(0.441)	(0.532)	(0.615)	(0.860)	(0.785)
Visual.Lateral(L)_DorsalAttention.IPS(L)	-0.14	-0.07	-0.23	-0.26	0.01	0.11	-0.11	0.16
	(0.575)	(0.773)	(0.342)	(0.231)	(0.960)	(0.615)	(0.615)	(0.491)
Salience.ACC_DorsalAttention.FEF(L)	-0.16	-0.07	0.09	-0.04	-0.19	-0.13	0.19	0.22
	(0.516)	(0.773)	(0.682)	(0.871)	(0.455)	(0.575)	(0.441)	(0.342)
DorsalAttention.FEF(R)_Language.IFG(R)	0.19	-0.01	0.04	-0.35	-0.05	-0.11	-0.10	-0.06
	(0.455)	(0.960)	(0.871)	(0.076)	(0.860)	(0.615)	(0.658)	(0.785)
FrontoParietal.LPFC(L)_FrontoParietal.PPC(L)	0.24	0.22	0.34	0.21	0.16	0.12	0.05	-0.10
	(0.340)	(0.372)	(0.079)	(0.382)	(0.516)	(0.609)	(0.849)	(0.658)
Parietal.LPFC(L)_Language.pSTG(L)	-0.17	0.03	-0.14	0.05	-0.05	0.13	0.10	0.12
	(0.491)	(0.882)	(0.542)	(0.860)	(0.849)	(0.575)	(0.671)	(0.609)

The table shows the Kendall's Tau correlation coefficients (with p-FDR values) between features of the final machine learning model using network rs-FC data. The significant correlations (after false discovery rate correction) were made bold with asterisk symbol (*). Note: Visual.Lateral (L), Visual.Medial = the left lateral visual network and the medial visual network, DorsalAttention.IPS (L), DorsalAttention.FEF (R) = the left intra-parietal sulcus node and frontal eye field node of dorsal attention network; Language.IFG, Language.pSTG = the inferior frontal cortex node and the posterior superior temporal gyrus node of language network; Frontoparietal.LPFC, Frontoparietal.PPC = the lateral prefrontal cortex node and the posterior parietal cortex node of frontoperietal network; Cerebellar.Anterior, Cerebellar.Posterior = anterior and posterior node of cerebellum; SensoriMotor.Lateral, SensoriMotor.Superior = the lateral sensorimotor network and the superior sensorimotor network; DefaultMode.MPFC, DefaultMode.PCC = the medial prefrontal cortex node and posterior cingulate cortex node of the default mode network; Salience.ACC, Salience.RPFC = the anterior cingulate cortex node and the rostral prefrontal cortex node of the Salience network. PSQI: Pittsburgh Sleep Quality Index; BAI: Becker's anxiety inventory; BDI-II: Becker's depression inventory version II; VAS: Visual Analog Scale; WPI: Widespread Pain Index; SSS: Symptom Severity Scale; FIQ: Fibromyalgia Impact Questionnaire; PPT (kg/cm2): Pain Pressure Thresholds measured in kg/cm2.

Table S5. Correlation coefficients between predictive structural features and clinical data

Variable	PSQI	BAI	BDI	WPI	SSS	FIQ	VAS	PPT
lVisPeri_ExStrSup_4	0.07	0.02	0.09	0.03	0.14	-0.05	0.09	-0.08
	(0.811)	(0.954)	(0.779)	(0.930)	(0.660)	(0.886)	(0.767)	(0.789)
lSomMotB_Aud_3	0.22	-0.03	-0.02	0.02	0.09	0.08	0.12	-0.04
	(0.407)	(0.947)	(0.954)	(0.954)	(0.767)	(0.786)	(0.682)	(0.925)
lSomMotB_S2_4	0.13	3e-3	0.04	-0.11	0.02	0.16	-0.16	0.26
	(0.660)	(0.982)	(0.923)	(0.719)	(0.954)	(0.585)	(0.585)	(0.282)
lDorsAttnA_ParOcc_1	0.07	-0.06	0.11	0.09	0.10	0.10	-9e-3	0.09
	(0.811)	(0.867)	(0.731)	(0.767)	(0.758)	(0.744)	(0.954)	(0.767)
lSalVentAttnA_FrMed_3	0.07	0.02	0.30	0.02	0.23	0.18	0.19	0.13
	(0.811)	(0.954)	(0.166)	(0.954)	(0.383)	(0.544)	(0.492)	(0.660)
lSalVentAttnB_PFCl_3	-0.06	0.13	0.13	0.14	0.13	-9e-3	0.20	0.09
	(0.873)	(0.660)	(0.660)	(0.660)	(0.660)	(0.954)	(0.480)	(0.767)
lDefaultB_PFCv_2	-0.04	0.11	0.04	0.24	-0.04	0.08	0.04	0.16
	(0.925)	(0.719)	(0.923)	(0.355)	(0.930)	(0.789)	(0.925)	(0.570)
rSomMotA_2	-0.01	0.03	0.04	0.03	0.08	0.16	0.14	0.08
	(0.954)	(0.947)	(0.925)	(0.947)	(0.789)	(0.570)	(0.660)	(0.789)
rDefaultC_IPL_1	-0.11	-0.05	0.08	-0.05	9e-3	0.14	0.13	0.34
	(0.744)	(0.886)	(0.789)	(0.918)	(0.954)	(0.660)	(0.660)	(0.100)

The table shows the Kendall's Tau correlation coefficients (with p-FDR values) between features of the final machine learning model using structural MRI data. No correlation was shown significant after false discovery rate correction. Note: lSalVentAttnB_PFCI_3 = the lateral prefrontal cortex regions of the left salience ventral attention network B; rDefaultC_IPL_1 = the right inferior parietal lobule node 1 of the default mode network C; lDefaultB_PFCv_2 = the ventral prefrontal cortex node 2 of the left default mode network; MisPeri_ExStrSup_4 = extra-striate superior node 4 ; lDorsAttnA_ParOcc_1 = parietal occipital node 1 of the left dorsal attention network A; lSalVentAttnA_FrMed_3 = the medial frontal node of the left salience ventral attention network A; lSomMotB_S2_4 = the S2 node 4 of the left sensorimotor network B; lSomMotB_Aud_3 = the auditory node 3 of sensorimotor network B ; rSomMotA_2 = the right sensorimotor network A node 2. PSQI: Pittsburgh Sleep Quality Index; BAI: Becker's anxiety inventory; BDI-II: Becker's depression inventory version II; VAS: Visual Analog Scale; WPI: Widespread Pain Index; SSS: Symptom Severity Scale; FIQ: Fibromyalgia Impact Questionnaire; PPT (kg/cm2): Pain Pressure Thresholds measured in kg/cm2.
